# Antarctic evidence for an abrupt northward shift of the Southern Hemisphere westerlies at 32 ka BP

**DOI:** 10.1038/s41467-023-40951-1

**Published:** 2023-09-05

**Authors:** Abhijith U. Venugopal, Nancy A. N. Bertler, Jeffrey P. Severinghaus, Edward J. Brook, Giuseppe Cortese, James E. Lee, Thomas Blunier, Paul A. Mayewski, Helle A. Kjær, Lionel Carter, Michael E. Weber, Richard H. Levy, Rebecca L. Pyne, Marcus J. Vandergoes

**Affiliations:** 1 GNS Science, Lower Hutt, 5010 New Zealand; 2 Antarctic Research Centre, Victoria University of Wellington, Wellington, 6012 New Zealand; 3 School of Physical and Chemical Sciences, University of Canterbury, Christchurch, 8041 New Zealand; 4 Scripps Institution of Oceanography, UC San Diego, La Jolla, CA 92093 USA; 5 College of Earth, Ocean and Atmospheric Sciences, Oregon State University, Corvallis, OR 97330 USA; 6 Physics of Ice, Climate and Earth, Niels Bohr Institute, University of Copenhagen, Juliana Maries Vej 30, 2100 Copenhagen, Denmark; 7 Climate Change Institute, University of Maine, Orono, ME 04469-5790 USA; 8 Institute for Marine and Antarctic Studies, University of Tasmania, 20 Castray Esplanade, Battery Point, TAS 7004 Australia; 9 Insitute for Geosciences, Department of Geochemistry and Petrology, University of Bonn, Bonn, 53115 Germany

**Keywords:** Palaeoclimate, Cryospheric science

## Abstract

High-resolution ice core records from coastal Antarctica are particularly useful to inform our understanding of environmental changes and their drivers. Here, we present a decadally resolved record of sea-salt sodium (a proxy for open-ocean area) and non-sea salt calcium (a proxy for continental dust) from the well-dated Roosevelt Island Climate Evolution (RICE) core, focusing on the time period between 40–26 ka BP. The RICE dust record exhibits an abrupt shift towards a higher mean dust concentration at 32 ka BP. Investigating existing ice-core records, we find this shift is a prominent feature across Antarctica. We propose that this shift is linked to an equatorward displacement of Southern Hemisphere westerly winds. Subsequent to the wind shift, data suggest a weakening of Southern Ocean upwelling and a decline of atmospheric CO_2_ to lower glacial values, hence making this shift an important glacial climate event with potentially important insights for future projections.

## Abstract (web summary)

Antarctic ice core records provide insights into past environmental conditions. Here, an abrupt, synchronous increase in dust from ice cores in Antarctica is identified that suggests a sudden equatorward shift of westerly winds and coincides with a reduction in atmospheric CO2.

## Introduction

During the last ice age and especially during Marine Isotope Stage (MIS) 3 (~60–27 ka Before Present (BP)), ice cores from Greenland and Antarctica document the presence of millennial-scale warm events known as Dansgaard—Oeschger (DO) events and Antarctic Isotope Maxima (AIM) respectively^1,2^. The evolution of these coupled climate events is primarily controlled by oceanic teleconnections driven by the variations in the strength of overturning circulation in the North Atlantic^3,4^, with far-reaching consequences such as changes in the Southern Ocean (SO) sea-ice conditions^5^. While the DO events were abrupt, the evolution of AIM events was gradual^1^. This implies the presence of a large ocean heat reservoir with high thermal inertia that modifies the signal in the Southern Hemisphere (SH)^3^. A recent study suggests that this heat reservoir could be the global interior ocean, located north of the Antarctic Circumpolar Current^6^.

However, the events also involved rapid and large-scale atmospheric re-organizations. In the SH, these changes are documented to cause a latitudinal displacement of Southern Hemisphere Westerly Winds (SHWW)^7,8^. The latitudinal positional of SHWW plays an important role in the rate of Southern Ocean (SO) upwelling^9,10^ and the amount of atmospheric dust entrainment from mid-latitude sources^11^. Consequently, changes in the SHWW during AIM-DO cycles are thought to have impacted atmospheric CO_2_ concentrations^12^, SO productivity^9^, and continental dust loading in Antarctica^5,11,13,14^. In addition, local insolation is also an important driver for climatic changes during MIS 3, particularly with its influence on SO conditions^15^. Sea-ice in some sectors of the SO started to advance by ~35 ka and expanded as far north as ~55°S by ~32 ka^16^, markedly before the Global Last Glacial Maximum (GLGM; 26.5–19 ka BP) or the local Antarctic LGM (29–19 ka BP)^17^.

Impurities measured in Antarctic ice cores such as non-sea salt calcium (nssCa^2+^- a proxy for continental dust) and sea-salt sodium (ssNa^+^ –a proxy for sea-ice/open-ocean changes) have been widely used to reconstruct environmental changes^18^. Here we present a new, well-dated, and decadally resolved record of ssNa^+^ and nssCa^2+^ from the Roosevelt Island Climate Evolution (RICE) ice core, an intermediate-depth ice core obtained from Roosevelt Island, West Antarctica (79.364^∘^ S, 161.706^∘^ W). We focus on the section of the record that captures a period between ~40 and 26 ka BP, spanning from mid-MIS 3 to early MIS 2.

Roosevelt Island is an independent ice dome grounded below sea level, located at the northern edge of the Ross Ice Shelf near the Ross Sea polynya, an important location for sea-ice generation^19^. This geographical positioning makes RICE a sensitive recorder for Ross Sea (southwest Pacific) climate variability and environmental conditions. The nssCa^2+^ record provides an opportunity to examine the dust transport and draw conclusions on the latitudinal position of SHWW, the ssNa^+^ record is used to infer the extent of large-scale sea-ice cover in the Ross Sea, and δD is used to understand the regional temperature and sea-ice conditions. Here we focus on a pan-Antarctic climate event that occurred at 32 ka BP and is marked by a significant change in RICE nssCa^2+^, RICE ssNa^+^, and RICE δD. We define the event based on RICE nssCa^2+^ and use ssNa^+^ and δD to understand the drivers.

In the second step, we compare RICE with existing ice cores and SH mid-latitude terrestrial records for spatial context, marine sediments, and climate model outputs to interrogate plausible hypotheses for the causes of this event and to investigate potential consequences, particularly on SO upwelling and global carbon cycle. RICE nssCa^2+^ and δD are published previously^19,20^ while ssNa^+^ is presented with this study.

## Results and discussion

### Glacial variability of continental and marine aerosols at Roosevelt Island

**Fig. 1:**
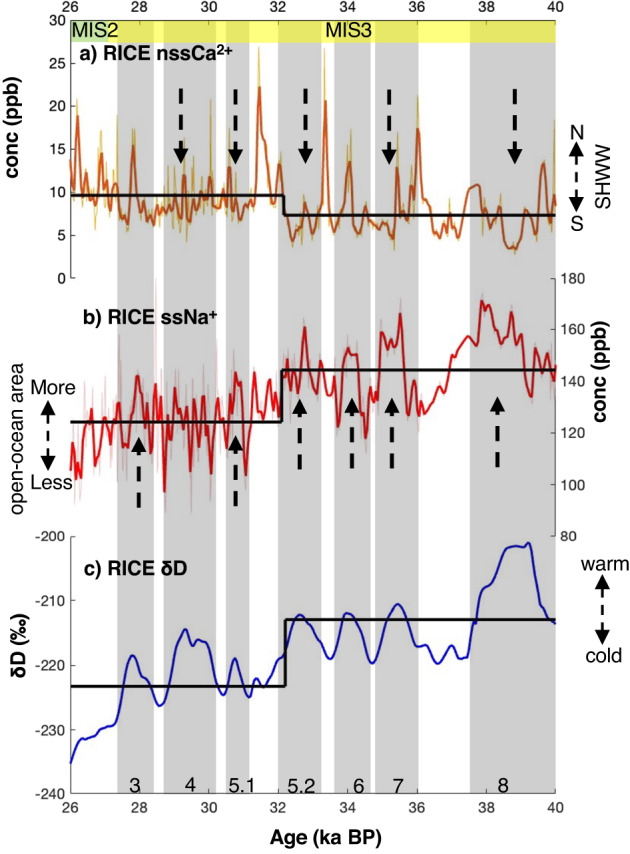
RICE glacial records between 40 and 26 ka BP. **a** nssCa^2+ 20^, **b** ssNa^+^ (this study), **c** δD^19^. Antarctic Isotope Maxima (AIM) events identified in RICE δD are shown in gray bars and are numbered at the bottom. Downward arrows in nssCa^2+^ indicate the lowering of concentration during AIM events and arrows pointing upwards in ssNa^+^ indicate an increase in concentration during AIM events. Black bold lines indicate the timing and magnitude of the mean shift. Thick brown and redline indicate a 50-yr average of the original nssCa^2+^ and Na^+^ data, respectively. δD is sampled on a 50-yr interval and smoothed using a 500-yr low pass filter. The latitudinal position of Southern Hemisphere Westerly Winds (SHWW) inferred from nssCa^2+^ concentration is shown using an arrow on the right side adjacent to the nssCa^2+^ plot. The direction of spatial change in the open-ocean area based on the Na^+^ level is shown by an arrow on the left side adjacent to Na^+^ plot. Higher δD values indicate atmospheric warming. Marine isotope stages (MIS) bracketed in this interval are shown using yellow and green bars at the top. The numbering of the events is based on WAIS Divide Ice Core (WDC)^31^. Source data are provided as a Source Data file.

At 32 ka BP, RICE nssCa^2+^ shows an abrupt transition across ~300 years from lower (~7.3 ppb) to higher mean values (~9.6 ppb; Fig. 1a; shown using the black line) and an overall increased variability. The change in mean values was determined using changepoint analysis^21,22^ (see Methods). During the glacial period, nssCa^2+^ measured in Antarctic ice cores is widely regarded as a proxy for Southern Hemisphere continental dust^5,11,13,23^. At RICE, the dominant source of Ca^2+^ has been shown to be marine during the Holocene^24^. In addition, minimal contributions from local Antarctic sources such as Marie Byrd Land is also possible^25^. We argue that the marine contribution would be modest during the glacial period as the ice-sheet extended until the continental shelf^26^ and SO sea-ice became more extensive^16^. Local Antarctic sources are also less likely to be prominent during the glacial period due to thick ice-cover. Hence, as we discussed in the previous study^20^, the dominant source of Ca^2+^ to Roosevelt Island during the glacial is likely to be dominated by terrestrial sources, originating from the SH mid-latitude sources, similar to cores of the Antarctic interior.

We use RICE δD (Fig. 1c; events shown using gray bars and numbers) to identify AIM events and we observe two types of changes in RICE nssCa^2+^. Type 1: AIM events 8, 7, 5.2, 5.1, and 4, are characterized by a reduction in nssCa^2+^ concentration (Fig. 1a; shown using downward pointing arrows). The drop is sharp for all the events, except AIM 4, where it appears to be more gradual. This concentration decrease has been observed in other Antarctic locations and is considered a characteristic of AIM events^5,11,13,23^. Type 2: During AIM events 6 and 3, we observe a centennial-scale peak (~500 years) in the nssCa^2+^ concentration (Fig. 1a). Such a feature is not documented in other sites and perhaps is specific to Roosevelt Island. We note that existing records are from high-elevation inland sites, while Roosevelt Island is a coastal site located at ~500 m elevation, perhaps a possible reason for this unique response.

During most millennial-scale climate variations, except AIM 4, ssNa^+^ in RICE increases with δD (Fig. 1b; shown using upward pointing arrows). During AIM 4 however, ssNa^+^ concentration appears to decrease. The increase in ssNa^+^ is delayed with respect to δD. For instance, during AIM 8 this delay is ~150 years. Changepoint analysis reveals that ssNa^+^ shifts to lower mean values at ~32 ka BP ( ~ 147.6 to 128.5 ppb) (Fig. 1b; shown using the black line), concurrent to the observed abrupt shift in nssCa^2+^. The source of ssNa^+^ archived in Antarctic ice cores can be traced either to open-ocean or sea-ice surfaces^27^. To infer the source of ssNa^+^ during the glacial, we calculated the SO_4_^2-^/Na^+^ ratio in our samples (Supplementary Fig. 1). Data that display values above the marine ratio (0.25) have been shown to originate from open-ocean bubble bursting^27^, while data below the marine ratio are thought to come from frost flowers formed on sea-ice^27^. The RICE SO_4_^2-^/Na^+^ ratio exceeds the threshold for the frostflower values, indicating an open-ocean origin for ssNa^+^ (Supplementary Fig. 1).

While it is possible that some portion of SO4^2−^ originates from biogenic activity, a correlation between non-sea-salt SO4^2−^ (nssSO4^2−^) and Methane Sulfonic Acid, a complex proxy for biological productivity^27^ shows a weak relationship (*r* = 0.26; *p* < 0.01) (Supplementary Fig. 2). This suggests the contribution of biogenic activity to total SO4^2−^ is minor and the main source is from the open-ocean. In addition to the higher SO4^2–^/Na^+^ ratio (>0.25), ssNa^+^ shows a decreasing trend from AIM 8 towards MIS 2/GLGM (Fig. 1b) when sea-ice is thought to have expanded^16,18^. This provides further evidence that ssNa^+^ record is sensitive to open-ocean area.

A spatial correlation analysis between RICE Na^+^ and the European Centre for Medium-Range Forecast (ECMWF) fifth-generation reanalysis products (ERA-5) sea-ice concentration^28^ for the period between 1951–2011 using Climate Reanalyzer (www.climatereaanlzyer.org) shows a statistically significant negative correlation between RICE Na^+^ and winter (June-August (JJA)) sea ice in the Ross Sea sector (Supplementary Fig. 3). The negative winter correlation suggests that, at least in modern times, RICE Na^+^ is sensitive to open-ocean area.

### Glacial climate variability at Roosevelt Island

We focus on δD in the well-dated section of RICE, from the time period 40–26 ka BP of the last glacial period (712.02 m to 725.84 m in depth) (Fig. 1c)^19^. δD is traditionally used as a proxy for local temperature^29^. However, other factors such as changes in elevation, sea-ice, moisture source and air mass trajectory also affect δD^30^. Even though the MIS 3 section is only 39–52 m above the bedrock (764.4 m), the comparison of RICE with high-resolution records from East and West Antarctica shows that AIM events are well captured (Supplementary Fig. 4) and that RICE δD is sensitive to regional to hemispheric temperature changes. Hence, we use δD as a proxy for regional temperature.

Amongst these millennial-scale events, it has been shown that AIM 8 and AIM 4 represent larger climate shifts as defined by their warm period duration of ~2000 years and temperature rise of ~3 °C^1^. AIM 7, 6, 5.2, 5.1, and 3 are identified as smaller climate fluctuations with a warm period duration of ~1000 years and a temperature change of ~1 °C^1^. Concurrent with nssCa^2+^ and ssNa^+^, the RICE δD record also shows a significant shift to more depleted values at ~32 ka BP (Fig. 1c; shown using the black line).

### Latitudinal re-organization of SHWW at ~32 ka BP

DO events in the North Atlantic caused opposite and lagged climatic responses in Antarctica through an oceanic teleconnection^31^. While DO-stadials led to gradual Antarctic warming, DO-interstadials resulted in gradual Antarctic cooling^1,3^. This lagged response in Antarctica points towards a large heat reservoir with high thermal inertia such as the global interior ocean that modifies the signal in the SH^6^. In addition, DO events also influenced SH climate through rapid atmospheric teleconnections^7,8^. At the onset of DO-stadials, the SHWW moves poleward^7,8^, away from potential source regions such as Patagonia, Australia, or New Zealand. This decreases the dust entrainment and results in a reduced deposition in Antarctica^11^. The reduction in atmospheric dust concentration is documented to precede the temperature change signifying the importance of dust transport. Overall, AIM events are characterized by reduced continental dust abundance^5,11,13,14^. This is observed in the RICE ice core with reduced nssCa^2+^ during most AIM events (e.g., during AIM 8, 7, 5.2, 5.1, and 4) (Fig. 1a). A poleward shift in the SHWW could also increase the amount of precipitation over the latitudes of the SO^32–34^. Such a change in the hydrological cycle can further contribute to dust removal through rainfall scavenging before it reaches deposition sites in Antarctica^32–34^.

**Fig. 2:**
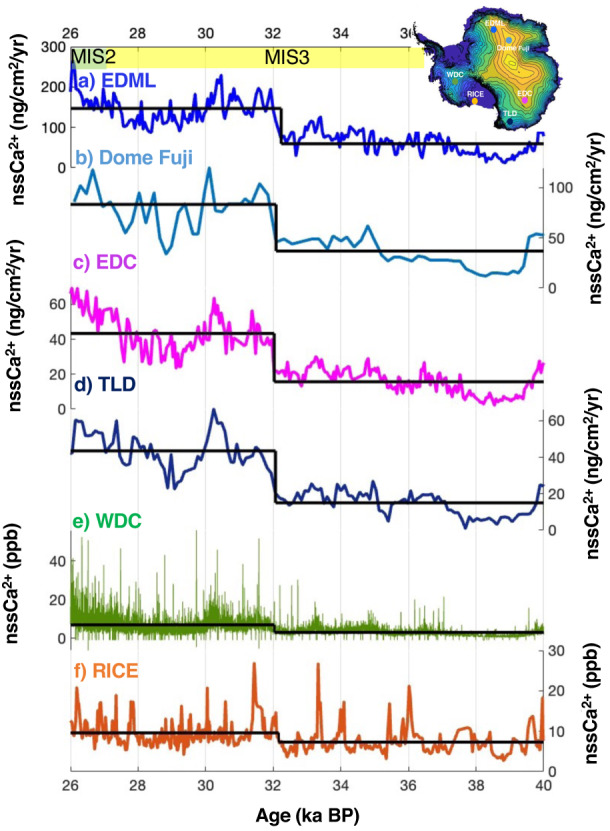
Glacial dust variability in Antarctica between 40 and 26 ka BP. **a** EPICA Dronning Maud Land (EDML) nssCa^2+^ flux^5^, **b** Dome Fuji nssCa^2+^ flux^44^, **c** EPICA Dome C (EDC) nssCa^2+^ flux^5,41^ **d** Talos Dome (TLD) nssCa^2+^flux^13^, **e** WAIS Divide Ice Core (WDC) nssCa^2+^ concentration^32^, **f** RICE nssCa^2+^ concentration^20^.All the records are presented on the WD 2014 age model based on the age-depth assignment provided in an earlier study at WDC^7^. Black lines indicate the timing and magnitude of the mean shift in each of the cores. The locations of the cores are shown in a surface elevation map inserted on the top right. Marine Isotope Stages (MIS) 3 and 2 are shown using yellow and green bars respectively at the top.

On a longer timescale, we observe a transition towards a higher mean dust concentration at ~32 ka BP (Figs. 1a, 2f). Building on our observations of Antarctic continental dust records during AIM events, an increase in the concentration at ~32 ka BP could suggest an equatorward migration in the mean position of SHWW. However, it is also possible that the elevated dust deposition is due to increased aridity in the source regions. Dust provenance studies using neodymium (Ne), strontium (Sr) and lead (Pb) isotopes in dust particles from East Antarctic cores such as EDC, Vostok, and Talos Dome suggest Patagonia as the major source region for dust during the glacial period^35,36^. Trajectory modeling suggests that in addition to Patagonia, continental dust may be transported to West Antarctic ice core sites from Australia and New Zealand^25,37^. Isotopic signatures of dust deposited in marine sediments from the South Pacific also confirm a non-negligible contribution from Australia during the glacial period^38^. Dust entrainment to the South Pacific from New Zealand is identified for the Holocene and it is suggested that it may have been more extensive during the glacial period due to favorable environmental conditions^39^. However, provenance studies are yet to confirm and quantify contributions from Australia and New Zealand in Antarctica during the glacial. MD07-2611 sediment record (geographic location shown in Supplementary Fig. 5) retrieved from the south of Australia provides information on Australia’s aridity. The fraction of quartz grains as well as titanium concentrations from MD07-2611-two independent proxies for regional aridity, show that the aridity in Australia began to intensify ~28 ± 0.6 ka BP^40^ (Supplementary Fig. 6), which is at least 3 ka after the observed increase in continental dust at RICE.

Over longer time periods, major environmental changes through changes in sea level and associated exposure of continental shelves as well as enhanced glacier erosion from expanding ice-sheet may be the key drivers for changes in atmospheric dust concentrations^41^. However, the global sea level did not start to fall until ~28 ± 0.2 ka BP^42^. Moreover, the expansion of the Patagonian ice sheet during MIS 3 was non-uniform both spatially and temporally^43^. While the ice sheet south of 48^ο^ S reached a maximum extent by 47 ka, the ice sheet between 48^ο^S and 38^ο^S grew to the maximum extent between 33 and 28 ka^43^. In addition, these changes in source conditions are known to evolve gradually and not abruptly. This suggests that source conditions are less likely to be the major factor responsible for the elevated continental dust concentration observed from ~32 ka BP.

### Comparison with other Antarctic ice core records

To assess the spatial extent of this change, we place the RICE record into a wider context by comparing the record with existing Antarctic ice core dust records (Fig. 2). We hypothesize that if the increased dust concentration at Roosevelt Island is linked to meridional transport, we would anticipate a) a similar increase across Antarctica, and b) that the shift occurs concurrently in all the records. Suitable well-dated records available from East Antarctica include: EDC^5,41^, EDML^5^, Dome Fuji^44^, and TLD^13^, and from West Antarctica is WDC^32^ (Fig. 2). The records are compared on a common age-scale (reference WD 2014 age scale) developed in earlier studies^7,19^. Concentration data were used for RICE and WDC, while flux was used for East Antarctic cores. Using the same changepoint analysis for all the records, we observe a remarkable synchroneity in the abrupt shift of nssCa^2+^ flux/concentration at 32 ka BP. We note that this increase is an Antarctic-wide phenomenon.

We also examine the insoluble dust concentration/flux data that are available from other Antarctic cores^11,45–48^ (Supplementary Fig. 7). The particle count data from RICE below ~100 m suffered from interferences and thus is not included. We also have omitted the EDML record from the analysis due to the sample gap between ~40–36 ka BP. The changepoint analysis reveals that dust flux in Dome Fuji and EDC and dust concentration in TLD increase abruptly at 32 ka BP. Vostok, despite its low resolution, still remarkably captures the transition from lower dust flux to higher dust flux around 32 ka BP. Based on our observations derived from AIM events that connect a poleward shifted SHWW with low dust concentration/flux, here we propose that this Antarctic-wide, sharp and substantial increase in atmospheric dust concentration at ~32 ka BP resulted from an equatorward migration of SHWW. In addition, decreased precipitation over the SO following the equatorward shift of the SHWW may also have contributed to elevated dust deposition due to reduced rainfall scavenging^32–34^.

To evaluate this hypothesis further, we investigate dust particle sizes in ice cores. EDC^49^ and EDML^45^ provide records of particle size changes which may carry information on the distance to the dust source (i.e. distal vs. proximal) (Supplementary Fig. 8). The particle size is considered generally within the range of finer particle size suggesting a long distance and higher tropospheric transport of dust^45,49^. Neither of the records displays any evident change before or after 32 ka BP. Hence, the grain size records remain inconclusive about the wind shift.

### Records for SHWW changes from SH mid-latitudes and SO

**Fig. 3:**
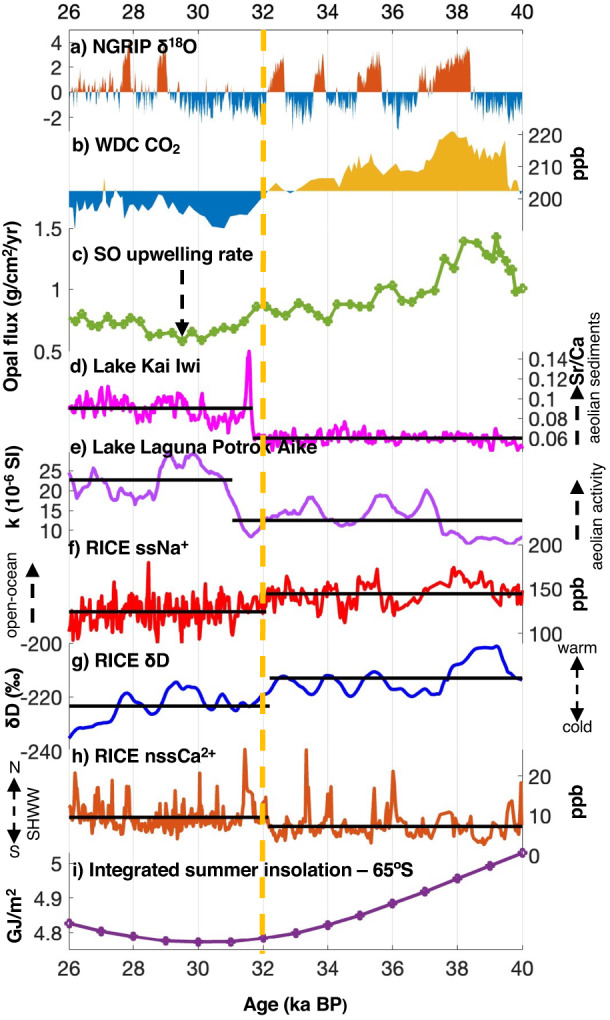
Records for glacial climate between 40 and 26 ka BP. **a** North Greenland Ice Core Project (NGRIP) δ^18^O (a proxy for Greenland temperature)^2^, **b** WAIS Divide Ice Core (WDC) CO_2_^23^, **c** Southern Ocean (SO) Opal flux (a proxy for SO upwelling)^9^, **d** Lake Kai Iwi Sr/Ca (a proxy for aeolian sediments)^50^, **e** Lake Laguna Potrok Aike k^51^ (a proxy for wind-derived dust), **f** RICE ssNa^+^ (a proxy for Ross Sea open-ocean area) (this study), **g** RICE δD (a proxy for temperature)^19^, **h** RICE nssCa^2+^ (a proxy for Southern Hemisphere Westerly Wind (SHWW) position)^20^, i) Integrated summer insolation curve^57^. Yellow dashed lines indicate the timing of the proposed SHWW migration at 32 ka BP. Lake Laguna Potrok Aike record is interpolated over 10-yrs and smoothed using a 500-yr window and the abrupt change is at 31±2 ka.

There are only a few well-dated, highly resolved terrestrial records from the SH mid-latitudes that extend beyond the LGM and hold information on the SHWW position^50^. A useful record comes from Lake Kai Iwi, located in Northland, New Zealand at ~36°S^50^ (geographic location is shown in Supplementary Fig. 5). Located in the SHWW belt (~30°S–60°S), the lake Kai Iwi Sr/Ca record derived from fine silt/clay sediments is interpreted as a wind proxy and shows an abrupt change towards higher concentration at ~31.7 ± 1.5 ka BP^50^ (Fig. 3d). This suggests an accelerated deposition of aeolian sediments, concurrent with dust records across Antarctica. The study links this abrupt shift in wind-derived sediments to an equatorward expansion of SHWW at ~32 ka BP^50^. Similarly, the magnetic susceptibility (k) record from Lake Laguna Potrok Aike in southern South America (geographic location is shown in Supplementary Fig. 5), a proxy for wind-derived dust displays a shift towards higher values at ~31 ± 2 ka BP, indicating an increased wind activity during this period^51^ (Fig. 3e).

Additional evidence comes from the reconstruction of the latitudinal position of the South Atlantic Subtropical Gyre (SASG) which suggests that the gyre was displaced northwards from at least 30 ka BP. This includes during Heinrich stadials 3 and 2, when the gyre is typically expected to shift poleward^52^. The latitudinal position of SASG is governed by the position of oceanic fronts namingly Subtropical, Subantarctic, and Polar Front^52^. In turn, the position of oceanic fronts is known to be sensitive to the latitudinal position of SHWW^53^. As the SASG was displaced northwards, this suggests the oceanic fronts were positioned relatively north, perhaps driven by the equatorward displacement of SHWW at 32 ka BP.

### Potential drivers of equatorward migration of SHWW at ~32 ka BP

The rapid re-organizations of SHWW during the last ice age are closely linked with DO cycles occurring in the North Atlantic^7,8^. While a DO-stadial results in poleward displacement of SHWW, during interstadials the SHWW shifts equatorward^7,8^. To assess the climatic condition in the North Atlantic, we examine the δ^18^O record (proxy for temperature) from the high-resolution North Greenland Ice Core Project (NGRIP) ice core^2^ (Fig. 3a). If the timing of the event at 32 ka BP were to coincide with a DO-interstadial, it could explain the equatorward shift in the SHWW. However, at 32 ka BP, stadial conditions prevailed in Greenland (Fig. 3a). Other Greenland ice core records also support this notion^54^. Millennial-scale changes in CH_4_ during the glacial are driven by the tropical climate^31^. As no major changes are observed in RICE CH_4_ at 32 ka BP^19^ (Supplementary Fig. 9), we suggest tropical drivers are unlikely to have had a major role.

We also assess tropical-extratropical teleconnections as potential drivers for the 32 ka event. The Pacific South American mode^55^ (Rossby wave train emitted by anomalous tropical deep convection during ENSO events) and Interdecadal Pacific Oscillation^56^ have been shown to be potent drivers of variations in Antarctic conditions. However, their impacts are asymmetric and regionally distinct, which stands in stark contrast to the remarkably concurrent dust shift at 32 ka across East and West Antarctica. Next, we investigate potential SH drivers.

Late MIS 3 involved a gradual reduction in local insolation in the SH^57,58^. Integrated summer insolation for 65°S, where summer is defined as days with insolation above a threshold of 275 W/m^2^, was at a minimum between ~32 and 28 ka, with a major reduction starting from ~39 ka (Fig. 3i)^57^. However, the insolation reduction is minor between ~33 and 32 ka (0.01 GJ/m^2^) and lower compared to earlier time intervals (e.g. 0.03 GJ/m^2^ between 39 and 38 ka). Therefore, it is less likely that the insolation changes had any direct influence on the abrupt SHWW wind shift.

Next, we examine sea-ice changes as captured in RICE. The records for RICE δD and ssNa^+^ show a significant change at ~32 ka BP, with δD showing a major depletion and ssNa^+^ exhibiting a lowered mean concentration (Fig. 1b, c). Previous studies examining the present-day conditions have shown that an increase in Ross Sea sea-ice can result in depleted signals in RICE water isotopes^59,60^. Moreover, decreased ssNa^+^ concentration at RICE suggests an increase in sea-ice in the Ross Sea. Interestingly, ssNa^+^ even appears to decrease during AIM 4, a major AIM event, suggesting more expanded and/or thicker sea-ice during this period. During modern times, an expansion in sea-ice in the Ross Sea has been shown to coincide with a decrease in snow accumulation at Roosevelt Island^59^. The snow accumulation rate, modeled based on a dynamic version of the Herron–Langway model (Supplementary Fig. 10), also shows a major change towards lower accumulation between ~34–32 ka BP^19^.

RICE δD, ssNa^+^, and accumulation rate changes together provide consistent evidence for an abrupt increase in sea-ice in the Ross Sea/South Pacific sector of the SO. Being one of the major SO regions for ocean-atmosphere heat exchange via the upwelling of warm and carbon-rich deep water and formation of Antarctic Bottom Water^19,59^, changes in sea-ice in the Ross Sea are of importance. Studies suggest that SHWW had migrated equatorward during recent cold periods such as the Antarctic Cold Reversal and Little Ice Age^53,61^. Climate models also support such a connection between the state of the SO and the latitudinal position of SHWW^10,58^. It is interesting to notice that this abrupt change is not captured in marine records (Supplementary Fig. 11), or ssNa^+^ in inland Antarctic ice cores (Supplementary Fig. 12). This indicates that the sea-ice change in the Ross Sea is regional. We are concluding that while the expansion of Ross Sea sea-ice appears to be linked to the 32 ka BP equatorward SHWW shift, its role in the event remains inconclusive.

The abrupt nature of the SHWW shift observed in the nssCa^2+^ data and sea-ice changes as observed in ssNa^+^ and δD points towards a critical threshold in the forcing. However, assessing the drivers of this shift is beyond the scope of this study. Focused modeling experiments using the data presented here are likely to help quantify contributions from both internal (e.g., feedback) and external factors (e.g., insolation changes).

### Potential climatic impacts of SHWW shift at 32 ka BP

The WDC record of CO_2_ shows a significant drop at 32 ka BP and remained at low values through the LGM (Fig. 3b; Supplementary Fig. 13a). This drop in atmospheric CO_2_ has been identified as noteworthy in numerous other studies^23,50^, particularly at WDC, where the drop in CO_2_ coincided with a major increase in Ca^2+^^23^. On millennial-scales, CO_2_ has been suggested to be influenced by SO processes such as the upwelling of carbon-rich deep water and change in Antarctic sea-ice extent^9,10,12^. A recent modeling effort also shows the state of North Atlantic Deep-Water formation and the flux of aeolian dust to the global ocean may also influence millennial-scale CO_2_ changes^62^. Opal flux to the ocean floor provides an independent record for the rate of SO upwelling and shows a major weakening of the overturning circulation between ~32–28 ka, with the lowest upwelling rate for the last glacial identified at ~30 ka (Fig. 3c; Supplementary Fig. 13b)^9^. It also appears that there is a transition in the upwelling rate into a suppressed mode after 32 ka (Fig. 3c; Supplementary Fig. 13b). A more poleward position of SHWW over the SO enhances the overturning circulation and CO_2_ release to the atmosphere, while its equatorward migration weakens the upwelling and CO_2_ outgassing^9,10,12^. Hence it is plausible that the drop in atmospheric CO_2_ at 32 ka BP is closely related to the proposed equatorward shift in SHWW which may have weakened the SO overturning circulation and reduced the CO_2_ outgassing to the atmosphere.

In addition to upwelling, changes in Antarctic sea-ice formation affect atmospheric CO_2_ concentration^23^. The RICE sea-ice records indicate an increase in regional sea-ice in the Ross Sea at 32 ka BP, which might have also assisted with the lowering of atmospheric CO_2_ concentration.

Finally, studies show evidence of maximum advance of multiple glaciers in New Zealand around ~31.5 ± 3 ka BP^63^. A variety of factors which include both climatic (temperature, precipitation, humidity, cloud cover) and non-climatic (e.g. local topography) factors can contribute to glacial advance and retreat and a single driver alone may not be sufficient to explain the changes^63^. However, it is possible that the increased frequency of cold and moist SO air masses associated with an equatorward shift of SHWW may have helped with the glacier advance, as noted during ACR in an earlier study, when SHWW is understood to have shifted equatorward^53^.

Coupled Model Intercomparison Project 6 models indicate that the position and strength of SHWW varies with the level of greenhouse gas forcing applied, where medium to high forcing leads to stronger and poleward shifted westerly winds, while low forcing causes the winds to weaken and shift equatorward^64^. As it is known that changes in the strength and/or position of SHWW can evoke large impacts on global and regional climate, understanding the changes in the past can provide crucial insights into the response of SHWW and its consequences under future climate scenarios.

In summary, Antarctic ice cores identify a continent-wide, abrupt shift in atmospheric dust concentration at ~32 ka BP. We provide evidence that links the change in dust loading to an equatorward shift of the SHWW. A reduction in the rate of SO upwelling is independently observed after the SHWW shift. This weakening of the overturning circulation in the SO may have contributed to the reported long-term drop in atmospheric CO_2_ following the 32 ka BP event. Advancing our understanding of the complex relationships and feedback between these important drivers and large-scale consequences is critical to improve future climate change projections.

## Methods

### Site description

The RICE project is a 9-nation collaboration that drilled and recovered the RICE ice core from Roosevelt Island, a local ice dome in the Ross Ice Shelf, West Antarctica^59^. The bedrock base of the dome lies ~214 m below sea level. The overlying ice has a surface elevation of ~550 m above sea level, with a total thickness of ~764 m at the crest. The drilling site (79.364° S, 161.706°W) has favourable temperatures (annual mean temperature = −23.5 °C) and high accumulation (22 ± 4 cm water equivalent per year (w.e.a^−1^)) to preserve high-resolution records of the climate and was chosen close to the ice divide to minimize layer distortion and advection at depth^59^. The upper 130 m of the core was recovered during the 2011–2012 field season, while the remaining section of the core (130–764.60 m) was retrieved during the following season.

### RICE 17 age model

The RICE 17 chronology is a combination of annual layer counting for the top 343 m, covering the past 2700 years^24^, and gas synchronization for the remaining section of the core^19^. For the time interval between 30.6 ka BP-1971 CE, RICE CH_4_, and δ^18^Oatm profiles are matched to the West Antarctic Ice Sheet (WAIS) Divide ice core (WDC) CH_4_ and δ^18^Oatm records using an automated algorithm. For older sections of the core, CH_4_ and δ^18^Oatm profiles in RICE were instead visually matched due to the limited resolution of the RICE CH_4_ record^19^. A dynamic version of the Herron–Langway model has been used to simulate the Δage and the RICE 17 ice-age scale is derived by adding Δage to gas-ages. RICE discrete and continuous CH_4_ and cumulative ice-age uncertainty for ~40–26 ka BP are shown in Fig. S9.

### Water stable isotope measurements

The stable water isotopic ratio (^2^H/^1^H, expressed as δD) in RICE samples were measured at the National Ice Core Laboratory, GNS Science, New Zealand using a continuous-flow melting system with an off-axis integrated cavity output spectroscopy (OA-ICOS) analyzer, manufactured by Los Gatos Research. The experimental setup has been discussed elsewhere^65^. Combined uncertainty for δD for 2 cm depth-averaged resolution is estimated as ±0.85‰^59^. The δD record discussed here is sampled at 50-yr intervals, calculated from decadal means, and smoothed with a 500-yr low pass filter^19^. Data was calibrated using in-house standards and normalized against the V-SMOW2/SLAP2 scale.

### Major ion measurements

Ca^2+^ and Na^+^ presented in this study were measured using a reagent-free Dionex Ion Chromatography System (ICS – 5000), with a 2 mm column and flowrate of 0.25 ml/min^20^. Samples were melted in a class-100 clean room facility at room temperature prior to analysis. Internal standards and an international standard, Atmospheric Environment Service (AES: Environment Canada), were inter-dispersed regularly within the samples for quality control. Precision is better than ~5% for both Ca^2+^ and Na^+^ based on internal standards. A total of 525 samples were measured for the period between ~40–26 ka BP.

### Calculation of non-sea salt calcium and sea-salt sodium

The non-sea salt fraction of calcium (nssCa^2+^) and sea-salt fraction of sodium (ssNa^+^) are calculated using the linear Eqs. (1) and (2), respectively.

1 $${{nssCa}}^{2+}={R}_{C}\,*\, \left(\left[{{Ca}}^{2+}\right]-{R}_{m}\left(\left[{{Na}}^{+}\right]\right)\,*\,{\left({R}_{C}-{R}_{m}\right)}^{-1}\right.$$

2 $${{ssNa}}^{+}=\left({R}_{c}\left(\left[{{Na}}^{+}\right]-\left[{{Ca}}^{2+}\right]\right)\,*\,{\left({R}_{c}-{R}_{m}\right)}^{-1}\right.$$

where, *R*_m_ and *R*_c_ are marine and crustal ratios of Ca^2+^/Na^+^ respectively^11^. We have used traditional values of *R*_c_ and *R*_m_ which are 1.78 and 0.038, respectively.

### Estimation of mean changes in chemical and isotope records

The mean shift in the chemical and isotopic records of ice cores mentioned in this study are identified using the *findchangepts* function in MATLAB. The function identifies the location in a record where a significant change in the mean occurs by employing a global parametric method^21,22^. According to this method, the function chooses a point in the record and splits it into two sections, computes the empirical mean for both sections, and calculates the total residual error. The function then moves the location of the division point, with the minimum number of samples between division points set to one, until the total residual error is at a minimum^21,22^.

Based on the algorithm, the change points are identified in RICE nssCa^2+^ at 32160 years BP, RICE ssNa^+^ at 32,096 years BP, and RICE δD at 32,200 years BP. The ice-age uncertainty (1σ) in RICE around 32,200 years BP is ~500 years (Supplementary Fig. 9)^19^.

Change points are also identified in other Antarctic nssCa^2+^ records: EPICA Dronning Maud Land (EDML), EPICA Dome C (EDC), Talos Dome (TLD), and WDC. Continental dust records from low-accumulation East Antarctic ice cores (EDML, EDC, and TLD) are presented as fluxes as accumulation in these locations is dominated by dry deposition, for which flux data more appropriately represent the atmospheric concentration^5,11,13^. In contrast, the accumulation in RICE and WDC are dominated by wet deposition^19,32,59^. For such regions, concentration values are more appropriate^19,32^. Synchronization of age-scales for these ice cores was achieved by identifying peaks of volcanic sulfur that exist in the ice-matrix and are placed on WD 2014 chronology^7^. Absolute ice-age uncertainty in each core is a combination of the uncertainty of the original WD 2014 chronology and the uncertainty associated with matching volcanic events and is ~40 years (1σ)^7^. Sample resolution may introduce additional uncertainty to the change point. To quantify this uncertainty associated with sample resolution, we performed a sensitivity study by resampling WDC nssCa^2+^ (sub-annual resolution) to the minimum resolution represented by Dome Fuji (~200 years) and computed the change point (Supplementary Fig. 14). We observe that the sample resolution can affect the timing of the change point by a maximum of ~30 years. The total uncertainty of the change point is thus a combination of age model uncertainty and the uncertainty associated with sample resolution. This is ~500 years for RICE and ~50 years for other ice cores. The change points for the mean shift are identified at 32,158 years BP at EDML, 32,080 years BP at Dome Fuji, 32,025 years BP at EDC, 32064 years BP at TLD, and 32,032 years BP at WDC. The timing of change points among the cores falls within the total uncertainty and any possible leads or lags remain indistinguishable. We, therefore, consider them to be synchronous within the uncertainty of the records.

To understand the sensitivity of the changepoint analysis, we carried out an additional test on RICE nssCa^2+^ using a different statistical variable (root mean square/variance). In addition to the mean, we also observe that root mean square/variance abruptly changes in RICE nssCa^2+^ at 32,160 years BP (not shown). This suggests that the change we observe at 32,160 years BP is significant. We also examined the change point by extending the record to 60 ka BP and observe that the changepoint would still be detected at 32160 years BP (not shown). The same approach of extending the record and performing the analysis was also employed on WDC nssCa^2+^. The changepoint was detected at ~32 ka BP (not shown), suggesting that the analysis is robust.

### Supplementary information

Supplementary Information

### Source data

Source Data

Source Codes

## Data Availability

Source data are provided with this paper. RICE Ca^2+^ used in the study are available in the PANGAEA Data Publisher (10.1594/PANGAEA.941412). RICE δD used in this study are available in the U.S. Antarctic Program Data Center (https://www.usap-dc.org/view/dataset/601359). PMIP 4 model outputs shown in the study are accessed from the following website— (http://hdl.handle.net/1959.4/100036). The data has also been shared in figshare data repository^66^. (10.6084/m9.figshare.23435966) Source data are provided with this paper.

## Code availability

Source code is provided along with this publication.
