# Supplementary material for: Antarctic evidence for an abrupt northward shift of the Southern Hemisphere westerlies at 32 ka BP
Source: Nat Commun. 2023 Sep 5;14:5432. doi: 10.1038/s41467-023-40951-1 (PMC10480229; doi:10.1038/s41467-023-40951-1)
Supplement: Supplementary file 1 — Supplementary Information [file 41467_2023_40951_MOESM1_ESM.pdf]

Supplementary Information

Antarctic evidence for an abrupt northward shift of the Southern Hemisphere westerlies at 32 ka BP

Abhijith U. Venugopal [1,2,3], Nancy A.N. Bertler[1,2], Jeffrey P. Severinghaus[4], Edward J. Brook[5], Giuseppe Cortese[1], James E. Lee[5], Thomas Blunier[6], Paul A. Mayewski[7], Helle A. Kjær[6,8], Lionel Carter[2], Michael E. Weber[9], Richard H. Levy[1,2], Rebecca L. Pyne[1], and Marcus J. Vandergoes[1]

[1]GNS Science, Lower Hutt, 5010, New Zealand

[2]Antarctic Research Centre, Victoria University of Wellington, Wellington, 6012, New Zealand

[3]School of Physical and Chemical Sciences, University of Canterbury, Christchurch, 8041, New Zealand

[4]Scripps Institution of Oceanography, UC San Diego, La Jolla, CA 92093, USA

[5]College of Earth, Ocean and Atmospheric Sciences, Oregon State University, Corvallis, OR 97330, USA

[6]Physics of Ice, Climate and Earth, Niels Bohr Institute, University of Copenhagen, Juliana Maries Vej 30, 2100 Copenhagen, Denmark

[7]Climate Change Institute, University of Maine, Orono, ME 04469-5790, USA

[8]Institute for Marine and Antarctic Studies, University of Tasmania, 20 Castray Esplanade, Battery Point TAS 7004, Australia

[9]Insitute for Geosciences, Department of Geochemistry and Petrology, University of Bonn, Bonn, 53115, Germany

Correspondence: Abhijith U.Venugopal (abhijith.ulayottilvenugopal@canterbury.ac.nz)

Supplementary Figures

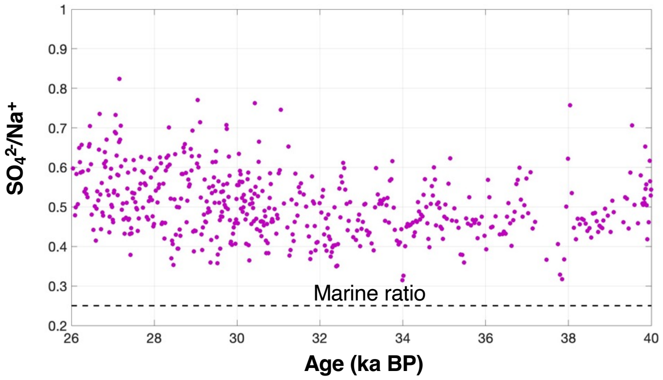

Supplementary Figure 1. Sulphate to sodium ration in RICE samples. Dashed black line indicates the marine ratio.

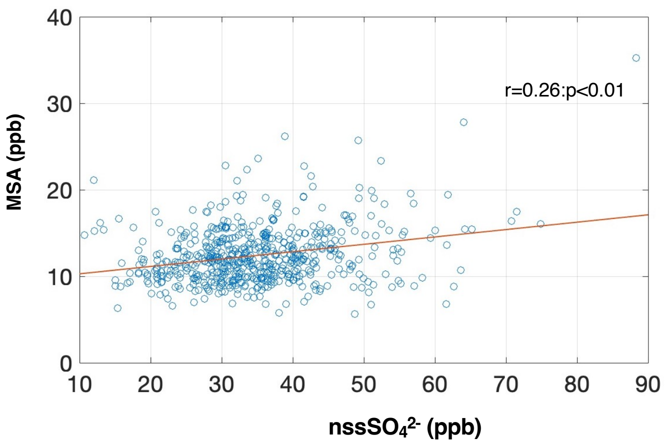

Supplementary Figure 2. Correlation between RICE $nssSO4^{2-}$ and MSA

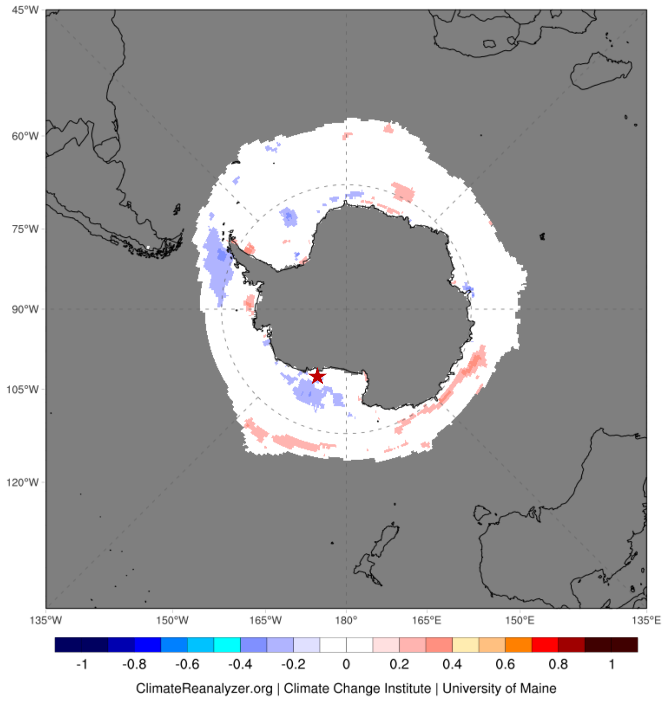

Supplementary Figure 3. Correlation between RICE Na^+ and ERA-5 sea-ice concentration[28] during winter (JJA) for the period between 1951-2011. Star symbol shows the location of Roosevelt Island. Correlation is shown above 90% confidence interval. Maps are created using Climate Reanalyzer (www.climatereanalyzer.org).

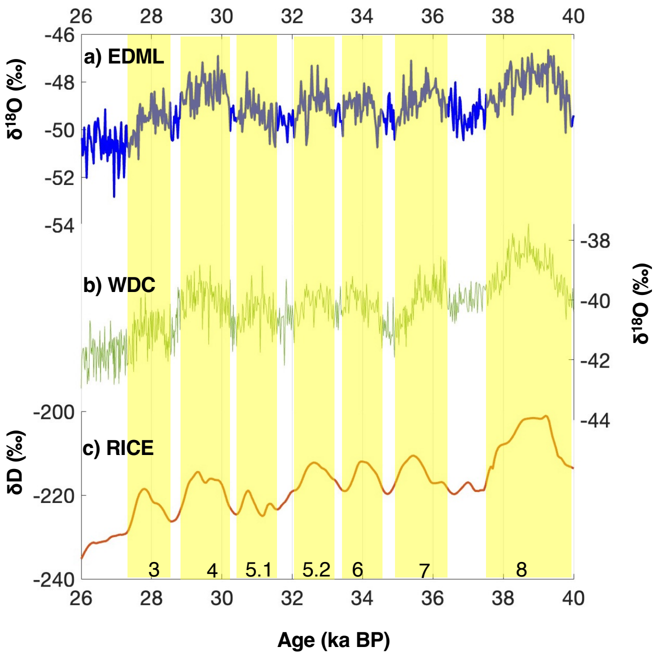

Supplementary Figure 4. Comparison of RICE isotope record with records from West and East Antarctica. a) EPICA Dronning Maud Land (EDML) $\delta^{18}O$[1], b) WAIS Divide Ice Core (WDC) $\delta^{18}O$[31], c) RICE δD[19]. AIM events are shown using yellow bars and are numbered at the bottom. The numbering is based on WDC core[31]. All records are on WD2014 chronology.

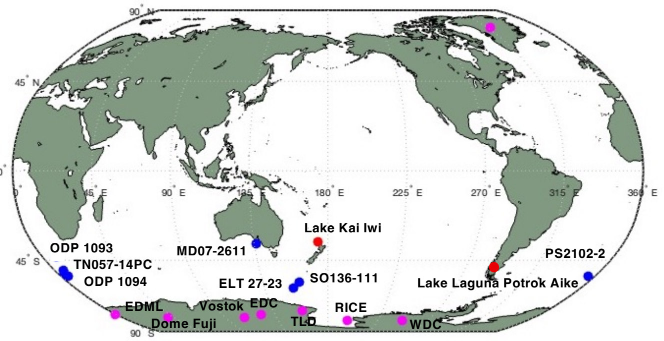

Supplementary Figure 5. Locations for the ice (magenta), marine (blue) and lake (red) cores discussed in this study. EDML [5,41], Dome Fuji[44] Vostok[67], EDC[5], TLD[13], RICE (this study), WDC[32], NGRIP[2], , ODP 1093[68] , TN057-14PC[9], ODP1094[68], MD07-2611[40], ELT 27-23[69], SO136-111[70], PS2102-2 [71], Lake Kai Iwi[50], Lake Laguna Potrok Aike[51]. The map is created using MATLAB with Antarctic Mapping Tools package[72].

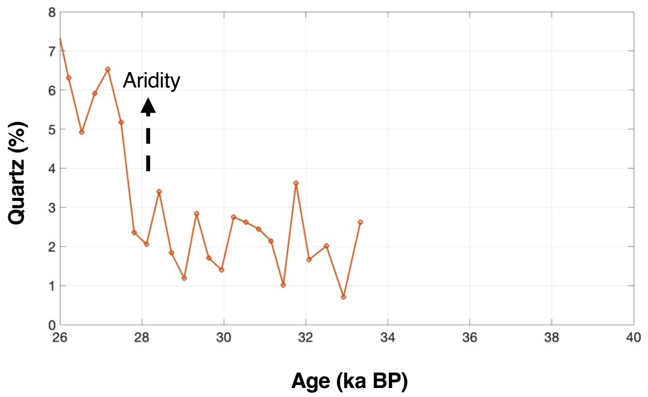

Supplementary Figure 6. Proxy record for aridity in Australia during the last glacial period[40]. Between ~33-25 ka BP, the record has an average resolution of ~300-400 years and age uncertainty of <600 years.

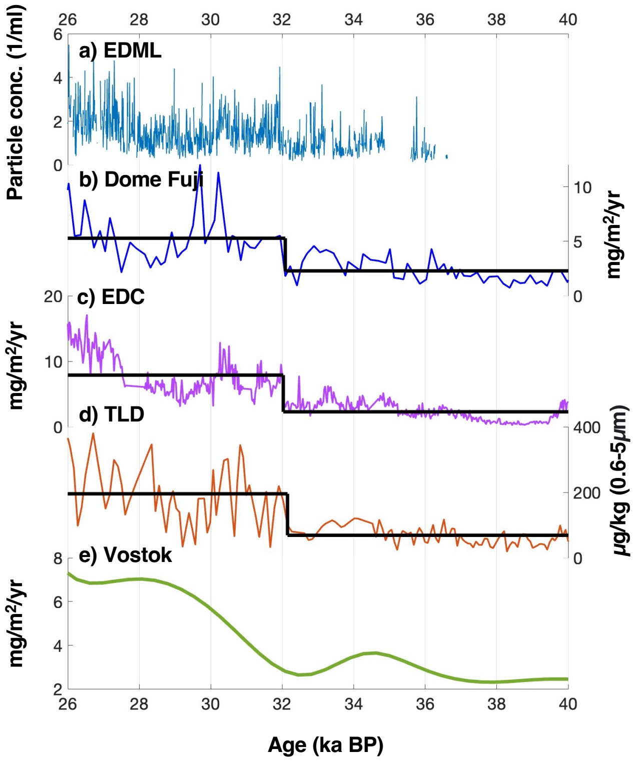

Supplementary Figure 7. Antarctic records for dust particle concentration/flux during the last glacial. a) EDML[45], b) Dome Fuji[48], c) EDC[11], d) TLD[46], e) Vostok[47]. Abrupt changes are identified through changepoint analysis. EDML was omitted from the analysis due to measurement gap between ~40-36 ka BP and Vostok due to low resolution. Black lines indicate the timing and magnitude of the mean shift in each of the cores.

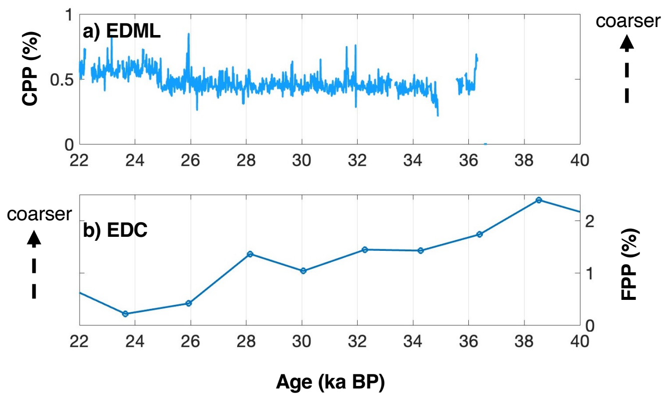

Supplementary Figure 8. Records for dust particle size in Antarctica. a) EDML[45], b) EDC[49]

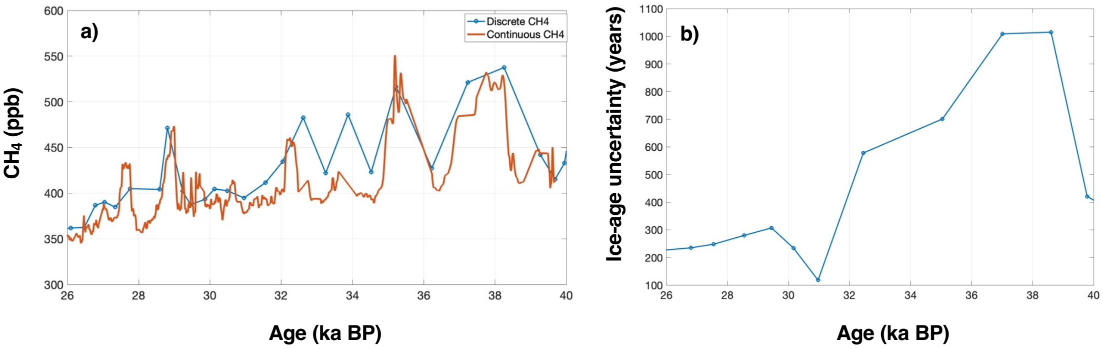

Supplementary Figure 9. a) RICE discrete (blue) and continuous (brown) CH_4 records, b) RICE age uncertainty between 40-26 ka BP[19].

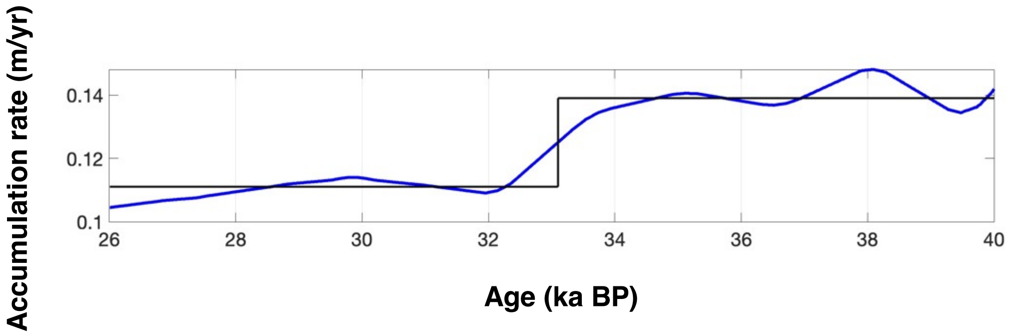

Supplementary Figure 10. Changes in RICE accumulation rate for the glacial period showing a distinct decrease between 34-32 ka BP[19].

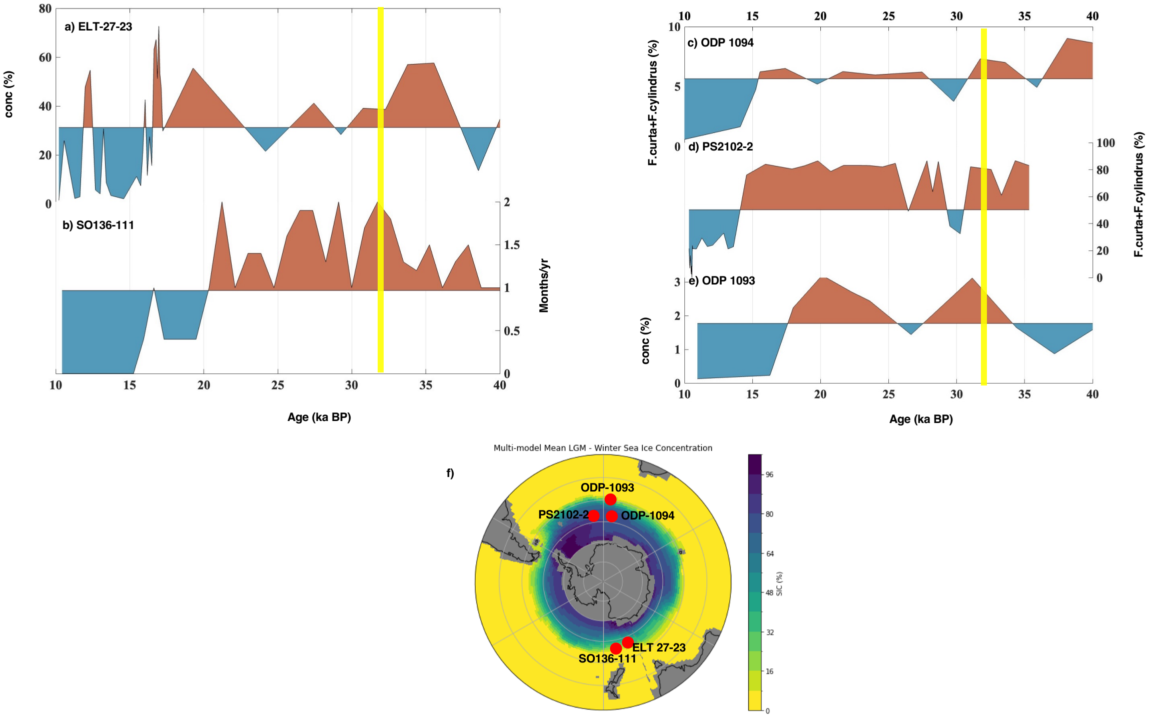

Supplementary Figure 11. Mid-late glacial sea-ice changes in the Southern Ocean. a) ELT 27-23 (Pacific sector)[69], b) SO136-111 (Pacific sector)[70], c) ODP 1094 (Atlantic sector)[68], d) PS2102-2 (Atlantic sector)[71], e) ODP 1093 (Atlantic sector)[68], f) Multi-model mean LGM winter sea-ice concentration from PMIP 4 simulations[73] (http://hdl.handle.net/1959.4/100036). The model simulations suggest that the cores (ELT-27-23, SO136-111, ODP 1094, ODP 1093 and PS2102-2) are located closer to the northern limit of the winter sea-ice in the SO and mostly outside of semi-permanent summer sea-ice (not shown) and therefore are suitable to capture major sea-ice changes.32 ka period is highlighted using yellow bar in plots a-e. Age uncertainties in the records are in the range of ~2.5-2.7 ka. Records do not show an abrupt change at 32 ka BP.

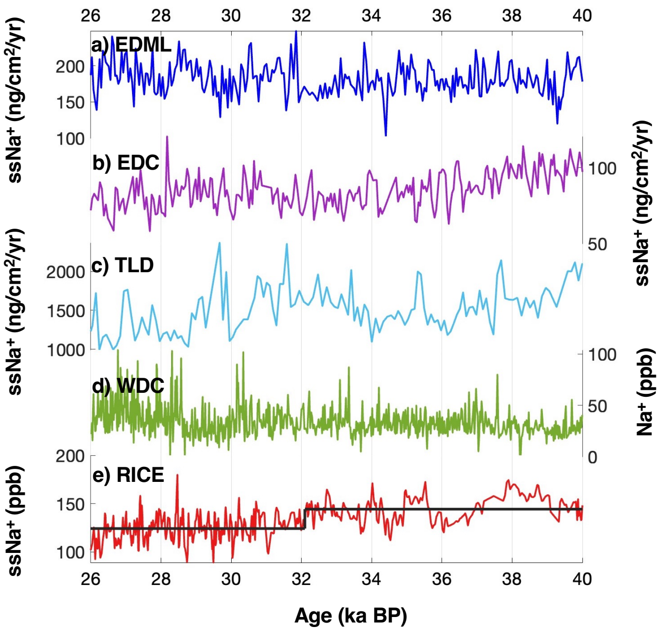

Supplementary Figure 12. Antarctic records for sea-ice changes during the last glacial. a) EDML $ssNa^+$ flux[5] b) EDC $ssNa^+$ flux[5], c) TLD $ssNa^+$ flux[13], d) WDC $ssNa^+$ concentration[23], e) RICE $ssNa^+$ concentration (this study). The change in the mean concentration in RICE at 32 ka is shown using black lines. All records are WD 2014 age model.

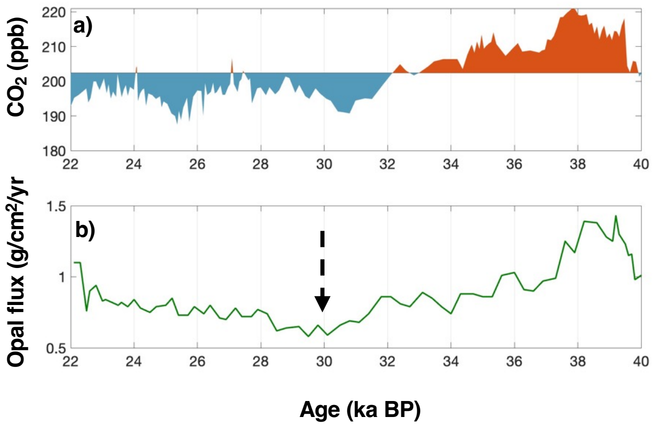

Supplementary Figure 13. Records for the last glacial CO_2 variability and Southern Ocean upwelling between 40-22 ka BP. a) WDC CO_2[23] , b) Opal flux record from TN057-14PC[9]. Arrow indicates the lowest period of upwelling.

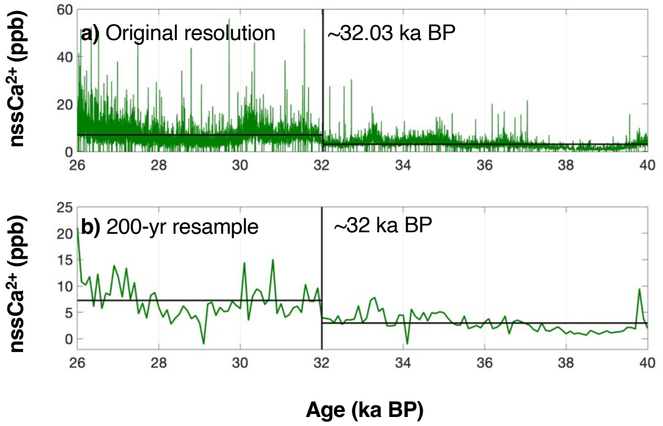

Supplementary Figure 14. Sensitivity of changepoint analysis to sampling resolution. a) WDC nssCa^{2+} concentration in original resolution (sub-annual), b) WDC nssCa^{2+} concentration resampled to 200-yr interval. The corresponding age to the changepoints are shown within the plot.

References

67 Petit, J. R. *et al.* Climate and atmospheric history of past 420000 years from Vostok ice core,Antarctica. *Nature* **399** (1999).

68 Mor, A. S. *et al.* Variable sequence of events during the past seven terminations in two deep-sea cores from the Southern Ocean. *Quaternary Research* **77**, 317-325 (2012).

69 Ferry, A. J. *et al.* First records of winter sea ice concentration in the southwest Pacific sector of the Southern Ocean. *Paleoceanography* **30**, 1525-1539 (2015).

70 Crosta, X., Sturm, A., Armand, L. & Pichon, J.-J. Late Quaternary sea ice history in the Indian sector of the Southern Ocean as recorded by diatom assemblages. *Marine Micropaleontology* **50**, 209-223 (2004).

71 Xiao, W. *et al.* Constraining the dating of late Quaternary marine sediment records from the Scotia Sea (Southern Ocean). *Quaternary Geochronology* **31**, 97-118 (2016).

72 Greene, C. A., Gwyther, D. E. & Blankenship, D. D. Antarctic mapping tools for MATLAB. *Computers & Geosciences* **104**, 151-157 (2017).

73 Green, R. A. *et al.* Evaluating seasonal sea-ice cover over the Southern Ocean at the Last Glacial Maximum. *Climate of the Past* **18**, 845-862 (2022).
