# Supplementary material for: Antarctic evidence for an abrupt northward shift of the Southern Hemisphere westerlies at 32 ka BP
Source: Nat Commun. 2023 Sep 5;14:5432. doi: 10.1038/s41467-023-40951-1 (PMC10480229; doi:10.1038/s41467-023-40951-1)
Supplement: Supplementary file 3 — Source Codes [file 41467_2023_40951_MOESM3_ESM.pdf]

READ_ME

Scripts for performing the changepoint analysis are provided in the ‘code section’ file.

Analysis is performed using *findchangepts* algorithm in MATLAB.

Data sets for the analysis are given in the excel file ‘Source Data’

Codes

Following MATLAB codes are used for changepoint analysis in RICE data. Data is made available as a supplementary file with this manuscript. Please refer to materials and methods section for more details on the analysis.

Changepoint should run with normal signal processing toolbox available in MATLAB.

Details can be found here - https://au.mathworks.com/help/signal/ref/findchangepts.html

```
# Code to find the single point in the data where mean most significantly changed
RICE_nssCa_cp=findchangepts(nssCa,'MaxNumChanges',1)
RICE_ssNa_cp=findchangepts(ssNa,’MaxNumChanges’,1)
RICE_dD_cp= findchangepts(dD,’MaxNumChanges’,1)
```
